# Nerve Decellularized Matrix Composite Scaffold With High Antibacterial Activity for Nerve Regeneration

**DOI:** 10.3389/fbioe.2021.840421

**Published:** 2022-01-28

**Authors:** Yan Kong, Di Wang, Qufu Wei, Yumin Yang

**Affiliations:** Key Laboratory of Eco-Textiles, Ministry of Education, College of Textile Science and Engineering, Jiangnan University, Wuxi, China

**Keywords:** nerve decellularized matrix, chitosan, antibacterial, nerve regeneration, schwann cells

## Abstract

Nerve decellularized matrix (NDM) has received much attention due to its natural composition and structural advantages that had proven to be an excellent candidate for peripheral nerve regeneration. However, NDM with simultaneous biocompatibility, promoting nerve regeneration, as well as resistant to infection was rarely reporter. In this study, a porous NDM-CS scaffold with high antimicrobial activity and high biocompatibility was prepared by combining the advantages of both NDM and chitosan (CS) in a one-step method. The NDM-CS scaffold possessed high porosity and hydrophilicity, exhibited excellent biocompatibility which was suitable for cell growth and nutrient exchange. Meanwhile, NDM-CS scaffold had a significant antibacterial effect on both *Escherichia coli* (*E. coli*) and *Staphylococcus aureus* (*S. aureus*), which could avoid wound infection during the repair process. In addition, the NDM-CS scaffold could support the growth and viability of Schwann cells effectively. Among them, the E2C1 group had the strongest ability to enhance proliferation, polarization and migration of Schwann cells among the three groups. The positive effect on Schwann cells indicated their ability in the process of nerve injury repair. Therefore, this NDM-CS scaffold may have potential prospects for application in neural tissue engineering.

## Introduction

Tissue engineering nerve scaffolds have aroused the interest of researchers because of their potential to replace autologous nerves (Jahromi et al., 2019). The ideal tissue engineering nerve scaffold mainly contains three elements: biomaterials, supporting cells and growth factors (Yi et al., 2019). As the most important factor in nerve scaffolds, suitable biomaterials have always been the key to investigate. Superior biomaterials could provide a suitable microenvironment for cell growth, as well as three-dimensional spatial structure, hydrophilic and antibacterial properties, etc. The three-dimensional structure of the nerve scaffold could provide enough pores and a staggered structure as growth space. The three-dimensional structure simulates the structure of the extracellular matrix, and the appropriate pores are conducive to the exchange of nutrients. Many tissue-engineered neural scaffolds based on natural or synthetic materials were composed of polymer materials (Ghane et al., 2021; Lu et al., 2021). Natural materials are more suitable than synthetic materials due to their biocompatibility, degradability, and potential hazards (Yang et al., 2007; Li et al., 2019). Another major problem after nerve scaffold implantation was bacterial infection (Tang et al., 2021a). Contamination during the procedure and recovery leads to increased use of antibiotics, which directly affects the outcome of nerve regeneration. Antibiotics or silver nanoparticles have been added to composite scaffolds as a slow-release agent to achieve an antimicrobial effect (Lan et al., 2014; Craciunescu et al., 2021). However, due to the short and unstable antibacterial effect of antibiotics or other antibacterial agents in the body, researchers are more inclined to look for a biological material with good antibacterial effect. Therefore, fabricating a nerve scaffold with good biocompatibility that could reproduce the natural nerve growth microenvironment and also possess natural antimicrobial properties is urgently needed in clinical practice.

The tissue decellularized matrix is an excellent natural material that could retain the natural extracellular matrix components intact. It is non-toxic, non-immunogenic and has reliable regeneration-promoting abilities, and thus was widely studied (Tao et al., 2021a; Rafaeva et al., 2021). Moreover, tissue decellularized matrix was considered as a candidate to replace autologous tissue in future clinical applications. It has been demonstrated that the deuterogenic materials derived from tissue decellularized matrix were used for the repair of bones (Qiu et al., 2020), skin (Xu et al., 2021), retina (Maqueda et al., 2021), nerves (Rao et al., 2021), muscles (Cheng et al., 2020), and other tissues and organs (Tao et al., 2021b; Wu et al., 2022). Moreover, the tissue decellularization matrix could induce direct differentiation of stem cells (Han et al., 2019). However, the decellularized matrix in per se did not have antibacterial properties and its effectiveness against postoperative infection was insufficient.

Chitosan (CS) with antibacterial function was a product of natural chitin deacetylation (Ke et al., 2021). CS has a positive charge, which can adsorb negatively charged bacteria and prevent bacterial transport across the membrane (Li and Zhuang, 2020). In addition, chitosan possesses excellent mechanical properties and provides stable mechanical strength for tissue engineering scaffold materials. CS had been certified as a valuable medical material and was widely used in various fields. The positive effects of chitosan and its degradation products on nerve injury repair have been confirmed (Wang et al., 2016; Boecker et al., 2019).

In this study, a facile method was developed to construct porous NDM-CS scaffolds that simulates the natural neural microenvironment with high antibacterial properties by a freeze-drying technique. The three-dimensional structure of the composite scaffolds were observed by scanning electron microscopy. Infrared spectroscopy and BCA Protein Assay Kit were used to analyze the composition of the scaffolds. The contact angle and porosity of the scaffolds also were tested. In addition, the antimicrobial properties of the scaffolds were evaluated by culturing the growth of *E. coli* and *S. aureus in vitro*. Cell culture experiment also demonstrated the excellent biocompatibility and ability of the NDM-CS scaffolds to promote the growth of Schwann cells. This study may provide a reasonable and reliable solution for future clinical applications of neural scaffolds.

## Materials and Methods

### Preparation of NDM

The nerves were decellularized according to the existing method in this study (Lin et al., 2018). Fresh sciatic nerves were decellularized by repeatedly shaking in 4% Triton X-100 for 6 h and in 4% sodium deoxycholate for 12 h at 4°C. Then the decellularized sciatic nerves were rinsed with ethanol for 1 h. Afterward, the nerve was divided into small segments for lyophilization. Finally, the lyophilized decellularized matrix was ground into a powder.

### Preparation of NDM-CS Scaffolds

**FIGURE 1:**
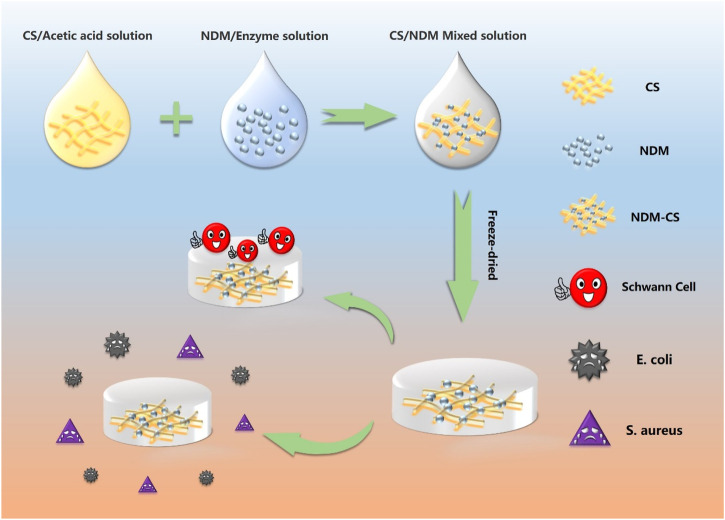
Schematic illustration of the preparation of NDM-CS composite scaffold and the experiment.

The NDM-CS hybrid lyophilized scaffold was prepared by the freeze-drying method. NDM and CS were dissolved separately and then mixed well before lyophilization. An 0.01 M hydrochloric acid solution of 2 mg/ml of pepsin was prepared which was used to dissolve the lyophilized neural decellularized matrix powder. The concentration of the decellularized matrix was 20 mg/ml dissolved with the acid solution at 37°C for 48 h. After the decellularization matrix was fully dissolved, 0.1 M sodium hydroxide solution was added to adjust the pH of the decellularization solution to neutral. 5% chitosan was fully dissolved in 2% aqueous acetic acid solution. Then three mass ratios of NDM:CS = 1:2, NDM:CS = 1:1 and NDM:CS = 2:1 (v/v) was prepared to fabricate mixed lyophilized scaffolds respectively. For ease of reference, the NDM:CS = 1:2, NDM:CS = 1:1 and NDM:CS = 2:1 were named as E1C2, E1C1 and E2C1 respectively. 400 μL of the mixed solution of different proportions were added to each well of the 24-well cell culture plate. After freeze-drying for 48 h, the three groups of mixed scaffolds were cross-linked with EDC:NHS = 1:2 ethanol solution for 24 h. And then the scaffolds were rinsed with 2% sodium hydroxide and PBS respectively. Finally, the scaffolds were lyophilized for 48 h again. Figure 1 shows the summary of the preparation process of the NDM-CS scaffolds and the overall experiment.

### Rheological Analysis

The viscosity properties of the NDM-CS solutions were tested to evaluate the differences in compositional changes between the three groups. The NDM solution and CS solution were mixed adequately at 4°C in different proportions firstly. Then 1 ml of each solution was pipetted out and added to the rheometer test platform. The experiments were conducted on a Rheometer (RheoWin MARS40, Thermo Fisher Scientific, USA) with parallel-plate (35 mm diameter) geometry at 25°C. Dynamic viscosity was tested at a 1 mm gap from 0.1 s^−1^ to 300 s^−1^ for 300 s.

### Morphological Observation

The morphology of the NDM-CS freeze-dried scaffolds was observed by scanning electron microscopy. 400 uL of three ratios of mixed solutions were added to the 24-well plate each well. The scaffolds were cut crosswise after being fully lyophilized. The scaffolds were observed from surface and cross-section respectively. The lyophilized scaffolds were fixed on the electron microscope carrier table, and observed after being gold plated on the material surface. Three parallel samples were set up for each group.

### Fourier Transform Infrared Analysis

Fourier transforms infrared (FTIR) spectra was used to analyze composition in scaffolds with tablets containing samples and KBr (KBr/sample ≈ 100). After the three NDM-CS scaffolds were lyophilized, a small portion of each scaffold was taken out and fully crushed with KBr (KBr/sample ≈100). After that, the mixed powder was pressed into slides under high pressure. The FTIR spectra were recorded from 4,000 to 500 cm^−1^. All tests have proceeded with humidity around 65% and at room temperature.

### Protein Content Detection

The BCA method was used to detect the content of NDM in the scaffolds. The NDM-CS solution was lyophilized in a 24-well plate, and then 3 ml of BCA working solution was added to each well. The scaffolds and BCA solution were incubated for 30 min at 37°C. At the end of the reaction, 200 μL of BCA reaction solution was aspirated from each group and the absorbance was measured at 562 nm.

### Porosity Detection

The ethanol substitution method was used to determine the porosity of NDM-CS scaffolds (Chen et al., 2020a). The total volume of ethanol origination was recorded as V_0_. The scaffolds were freeze-dried in 24-well plate with 400 μL solution, then were immersed into the ethanol and put under the pressure of 0.08 MPa for 1 min. The total volume of ethanol and the scaffolds at this time was recorded as V_1_. Then the scaffolds were taken out of the ethanol and the remaining ethanol was recorded as V_2_. The porosity was calculated by the following formula:

$$\left({\text{V}}_{\text{0}}-{\text{V}}_{2}\right)/\left({\text{V}}_{1}-{\text{V}}_{2}\right)\times 100\%$$

### Hydrophilic Property

The hydrophilicity of NDM-CS scaffolds was detected by the contact angle. The smaller the contact angle, the better the hydrophilicity of the material. The three groups of NDM-CS composite scaffolds were lyophilized in 24-well plates. Then all the NDM-CS composite scaffolds were taken out from 24-well plates and placed on the test bench of the contact angle instrument (JYPHa, Chengde, China) under the dry conditions. The contact angles of the scaffold were measured by the wetting of the droplets on the scaffolds. Five parallel samples were set for each group of materials. All samples were performed at room temperature and around 65% humidity. In this study, the contact angles of droplets were examined at the beginning of contact with the material and after 30 s of contact respectively.

### *In Vitro* Antibacterial Experiment

The antibacterial experiment *in vitro* was referred to the common experimental methods (Tang et al., 2021b). 100 μL of *E. coli* and *S. aureus* (≈10^4^ CFU/ml) (China Microbial Culture Collection, China) were incubated with the freeze-dried scaffolds at 37°C for 4 h. Then 900 μL of phosphate-buffered saline (PBS) was added to resuspend *E. coli* and *S. aureus*. 100 μL of the resuspended *E. coli* and *S. aureus* solution were spread on a solid LB agar medium plate respectively. The colonies formed were counted after culturing for 24 h. The calculation formula of bacterial kill rate was:

$$\text{Kill}\%{=\text{C}}_{\text{0}}-\text{C}/{\text{C}}_{\text{0}}\times 100\%$$

C indicates the CFU of the scaffolds and C_0_ indicates the CFU of the control group inoculated on a blank plate.

### Cytotoxicity Test

Firstly, each group of scaffolds was sterilized by ultraviolet light and 75% ethanol for 30 min respectively. The scaffolds were immersed in basal medium for 48 h under 37°C sterile conditions. Then 10% fetal bovine serum (FBS, Gibco, USA) was added into the extraction medium of each group for culturing L929 cells. 1 × 10^4^ cells/mL were cultured in each well of the 96-well plate with 200 μL. And there were five replicate wells for each group. The normal culture medium group served as the control group. After being cultured for 24 h in a cell incubator, 20 μL MTT was added to each well and then incubated for another 4 h in the dark condition. Then all the culture solution was discarded and 200 μL dimethyl sulfoxide (DMSO, Sigma-Aldrich, USA) was added to incubate for 30 min. Then the absorbance at 570 nm of each well was measured by an enzyme-labeled instrument.

### The Viability and Morphology of Schwann Cells

The RSC96 cells in this experiment were obtained from the Center for Excellence in Molecular Cell Science, Chinese Academy of Sciences. The DMEM infusion of NDM-CS scaffolds was used to culture Schwann cells. Firstly, the DMEM infusion of three scaffolds was added with 10% FBS and 1% penicillin-streptomycin antibiotic to configure the complete culture medium. Then RSC96 cells were resuspended with the complete medium above. 0.5 ml of RSC96 cell suspension was added dropwise to each of the three sets of NDM-CS scaffolds and incubated in a cell incubator.

The viability of RSC96 cells was assayed by the cck-8 method after 1 and 2 days of incubation. The cell culture solution was discarded firstly and 1 ml of cck-8 working solution (cck-8: complete medium = 1:10) was added to each well. Then the incubation was continued for 4 h. Finally, the absorbance value at 450 nm of each group of cck-8 working solution was detected.

The samples were washed once with PBS and the cells were fixed with 4% paraformaldehyde for 4 h subsequently. Fluorescent blocking solution was added overnight for 4 h after PBS rinsing. Afterward, RSC96 was specifically labeled with fluorescent dyes phalloidin and observed under a microscope.

### Migration of Schwann Cells

The study used Ibidi chambers to form stable wounds to study the migration of Schwann cells. Sterile ibidi chambers adhered to the bottom of cell culture plates. Afterwards, 80 μL of RSC96 cell suspension (complete medium) was added to the ibidi chambers. The ibidi chambers were removed after being incubated in a cell culture incubator for 12 h. Then serum-free DMEM was added to the wells. Photographs were taken and recorded as the 0 h at this time. After that, pictures were taken after 6, 12 and 24 h of incubation respectively.

### Statistical Analysis

Statistical analyses of all data (mean ± SEM) were performed using GraphPad prism 8. Comparisons across three groups and two groups used one-way analysis of variance (ANOVA) testing and two-tailed *t*-test where a *p*-value < 0.05 was considered significant respectively. Sample sizes are indicated in figure legends.

## Results and Discussion

### Viscosity of the Mixed Solution

**FIGURE 2:**
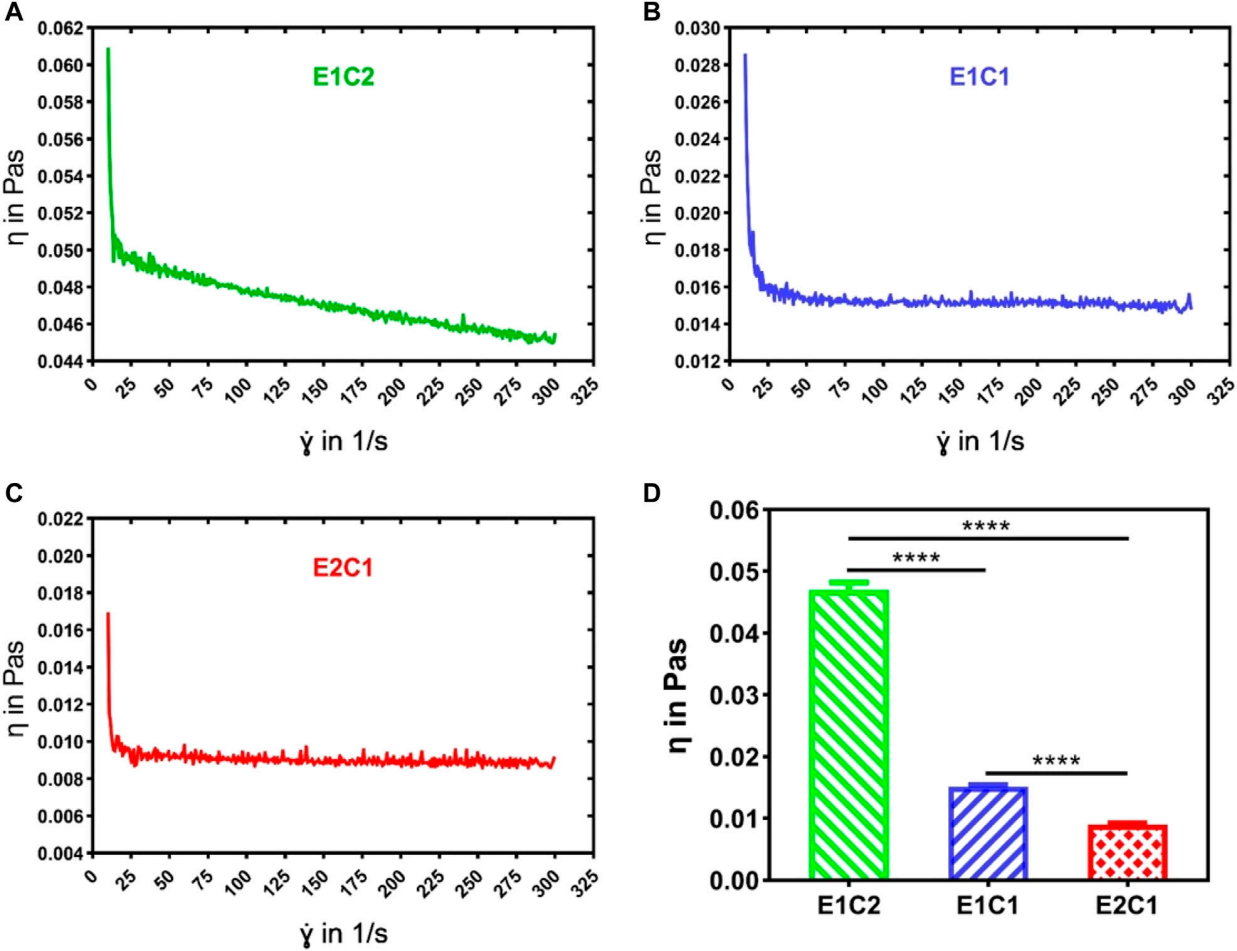
The viscosity curves of the three mixed solutions were detected by the rheometer: **(A)** E1C2, **(B)** E1C1, **(C)** E2C1, **(D)** Statistical results of the viscosity of the three mixed solutions. ****p < 0.0001.

The viscosity of the mixed solutions with different ratios was analyzed by rheometry (Figures 2A–C). The results revealed that the viscosity of all mixed solutions showed property of shear-thinning as the shear rate increased. The E1C2 group showed the greatest degree of shear-thinning, while the E2C1 group showed the least degree of shear-thinning. This phenomenon was mainly caused by chitosan. The shear-thinning behavior was more obvious with the increasing amount of chitosan solution. Figure 2D shows the average viscosity of the three mixed solutions. It showed that the viscosities of the E1C2, E1C1 and E2C1 groups were 0.047, 0.015 and 0.009 Pa·s respectively. There were significant differences among them. The trend of viscosity changes was consistent with the trend of shear-thinning which indicated that the effect of chitosan solution on viscosity change was significant. Also, the phenomenon reflected the good mechanical properties of chitosan, which played a good role in supporting the stability of the scaffold.

### Morphological Observation

**FIGURE 3:**
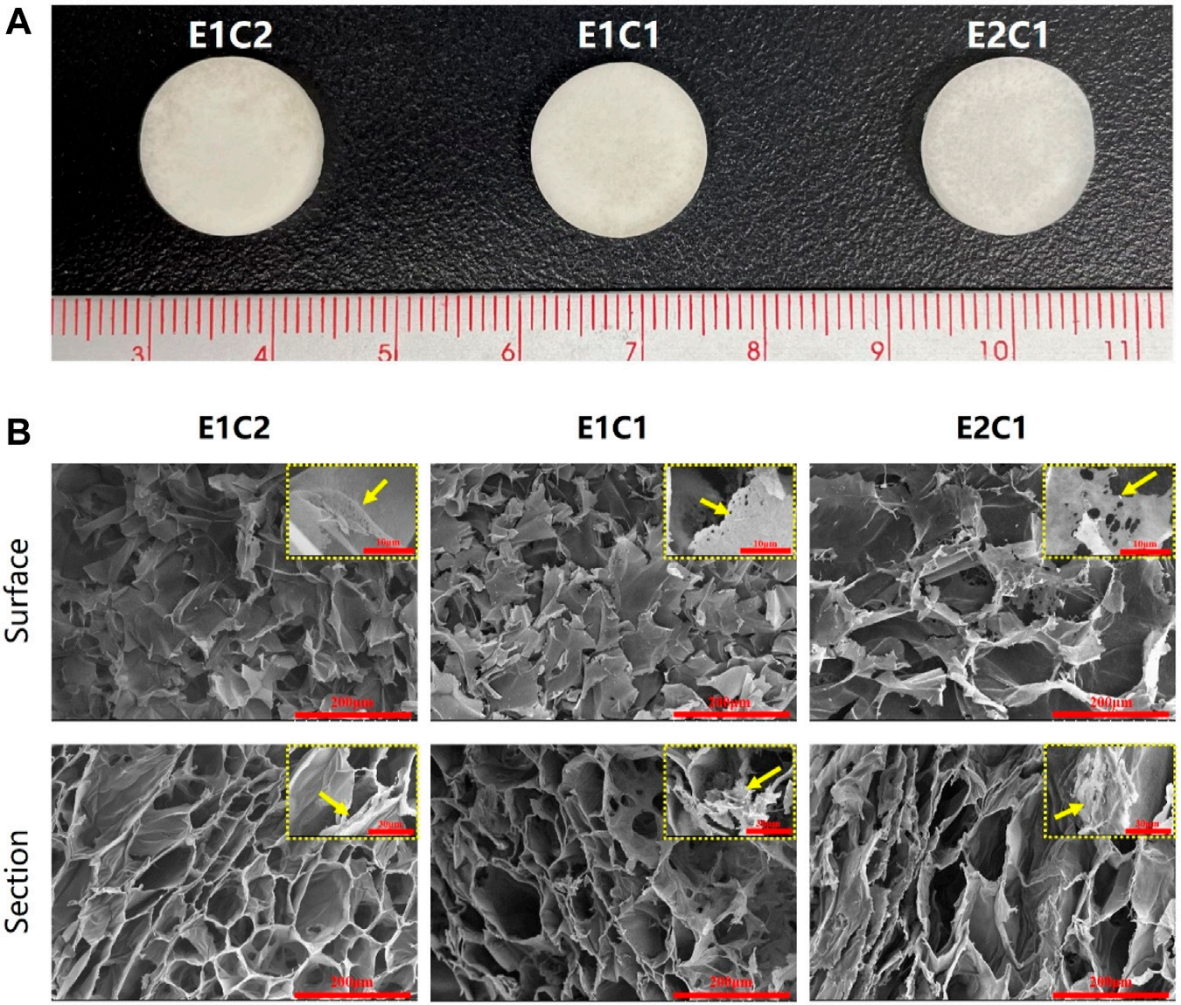
Observation of the NDM-CS scaffolds. **(A)** The macroscopic state of NDM-CS composite scaffolds. **(B)** Scanning electron microscope observation of the surface and cross-section of NDM-CS scaffolds.

The three groups of mixed scaffolds with different ratios did not differ from each other under the macroscopic and microscopic state (Figure 3). Figure 3A showed the macroscopic morphology of three groups of NDM-CS scaffold. All groups appeared white spongy. Scanning electron micrographs allowed clearer observation of the morphology of the three groups of scaffolds. Both the surface and the cross-section showed a clear pore structure under the observation of scanning electron microscope (Figure 3B). All groups showed a lamellar structure of lyophilized chitosan on the surface which were densely and uniformly distributed. And the cross-section could be observed which possessed a honeycomb-like three-dimensional pore structure. In the magnified SEM photographs, it could be found that there were thin layers or agglomerates with small size and pores between the chitosan lamellar structures or on the surface of each group of scaffolds (pointed by the yellow arrow). This fine structure which was different from chitosan could be observed in the cross-section of the scaffolds too. Moreover, this structure increased with ascending NDM concentration. The agglomerates were supposed to be formed after lyophilization of neural decellular matrix protein. This result indicated that the neural decellular matrix was successfully cross-linked with chitosan.

### FTIR Analysis

**FIGURE 4:**
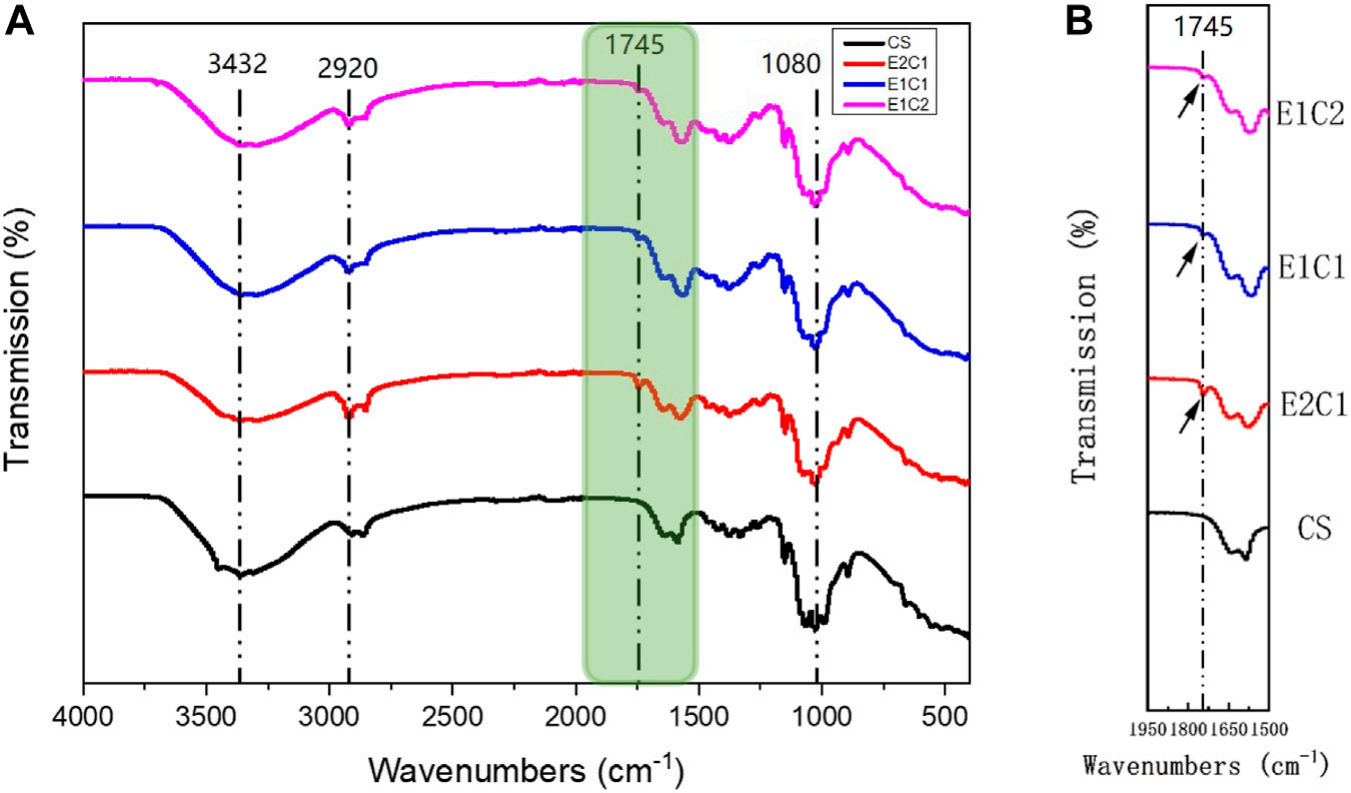
FTIR analysis of the scaffolds. **(A)** Detection curve from 500 cm^−1^ to 4,000 cm^−1^. **(B)** Enlarged view of the green area in the FTIR curve.

The changes in the composition of the hybrid scaffold were analyzed by FTIR detection. Figure 4A shows the FTIR curves of the four groups of scaffolds. The pure CS scaffold was used as a control. The FTIR curves of the four materials were almost identical. In all groups, the peak of the stretching vibrations of the O-H and N-H groups could be found near 3,432 cm^−1^. The C-H stretching band and the stretching vibrational band of C-O could also be observed near 2,920 cm^−1^ and 1,080 cm^−1^ respectively. However, besides the CS group, the other three hybrid scaffolds had a typical distinct C=O stretching peak of dECM at around 1745 cm^−1^ (Xu et al., 2021). In addition, the peak area of C=O stretching became more and more obvious as the NDM concentration increased (Figure 4B). Such results indicated that the NDM-CS scaffold did contain the NDM protein component. And the change of the characteristic peak of C = O also reflected the change of the concentration of NDM contained in the scaffold. Thus, it was verified that the neural decellularized matrix protein and chitosan were successfully bound together.

### NDM Protein Content

**FIGURE 5:**
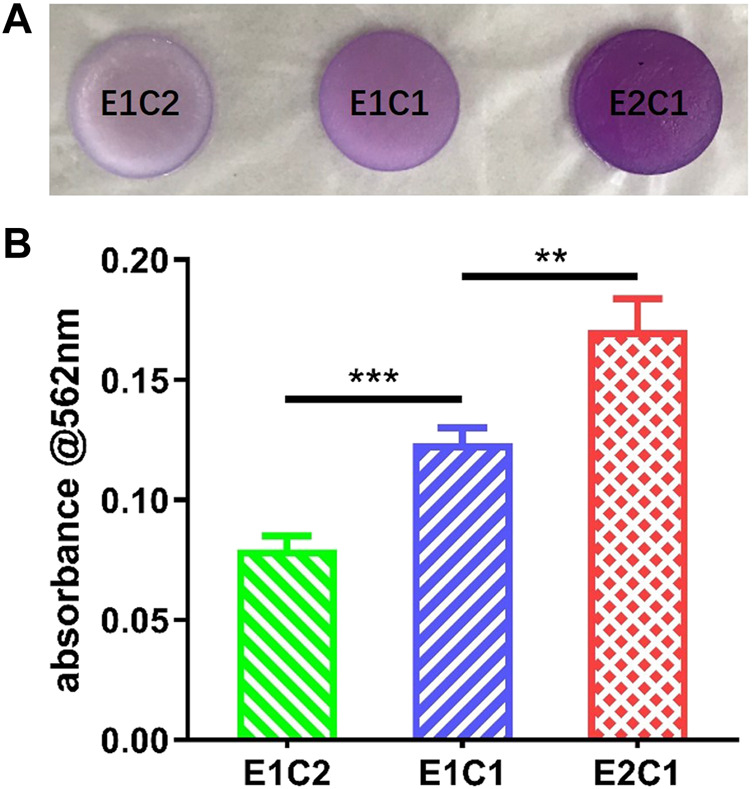
**(A)** Macrophotographs of different scaffolds after BCA reaction. **(B)** The OD value of the BCA reaction solution at 562 nm **p < 0.01, ***p < 0.001.

The infrared analysis only gave a qualitative insight into the presence of neural decellular matrix in the hybrid scaffolds. Protein concentration assay was performed to further verify the changes in the NDM ratio in the three sets of scaffolds (Dong et al., 2020). Figure 5A demonstrates the formation of purple complexes in the three groups of hybrid scaffolds after adsorption of the protein reaction solution. It was shown that the E1C2 group had the lightest purple color while the E2C1 group had the darkest purple color. In addition, the absorbance comparison results of the protein reaction solution at 562 nm for the three groups of materials showed the same trend (Figure 5B). The absorbance value of E1C2 was the smallest at 0.079 while the absorbance of E2C1 group was the largest at 0.17. The absorbance of E1C1 group was 0.12 which was between the other two groups. And there was a significant difference between all three groups. These results proved that the concentration of NDM was completely different in the three hybrid scaffolds. the amount of NDM in E2C1 was the most and twice as much as that in E1C2. The amount of NDM in E1C1 was 1.5 times more than that in E1C2. This was fully consistent with the ratio change of the three hybrid scaffold designs.

### Porosity of the Scaffolds

Porosity refers to the percentage of the pore volume in the total volume of the material in its natural state. A certain porosity is conducive to the exchange of nutrients and beneficial to cell growth and hyperplasia (Zhu et al., 2015; Ardeshirylajimi et al., 2018).

**FIGURE 6:**
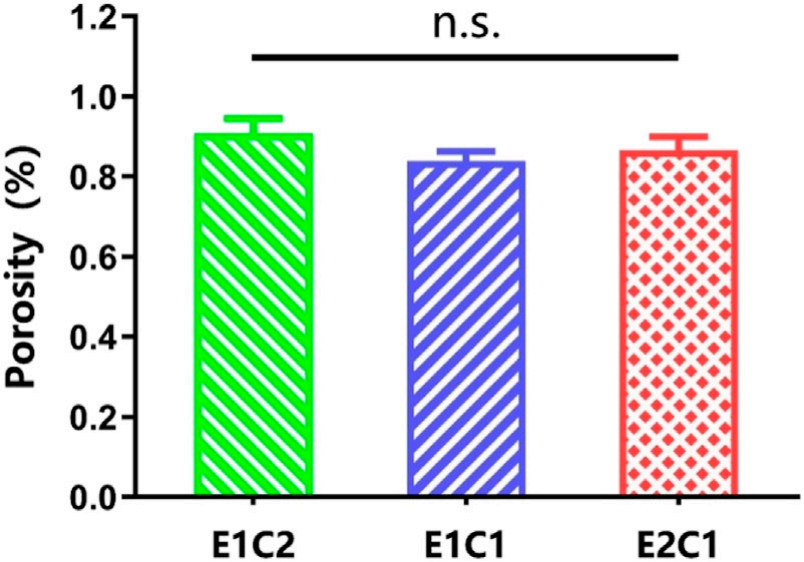
The porosity of the scaffolds. n.s.: *p* > 0.05.

The results in Figure 6 shows the porosity of the three groups of hybrid scaffolds. The porosity of all three groups was above 80% and did not differ from each other which indicates that although there were slight differences in the three groups of scaffold components, it did not affect the overall porosity. All groups of scaffolds had enough three-dimensional pores to meet the infiltration and exchange of nutrients and air, which could provide a suitable growth environment for cells.

### Wettability Analysis

**FIGURE 7:**
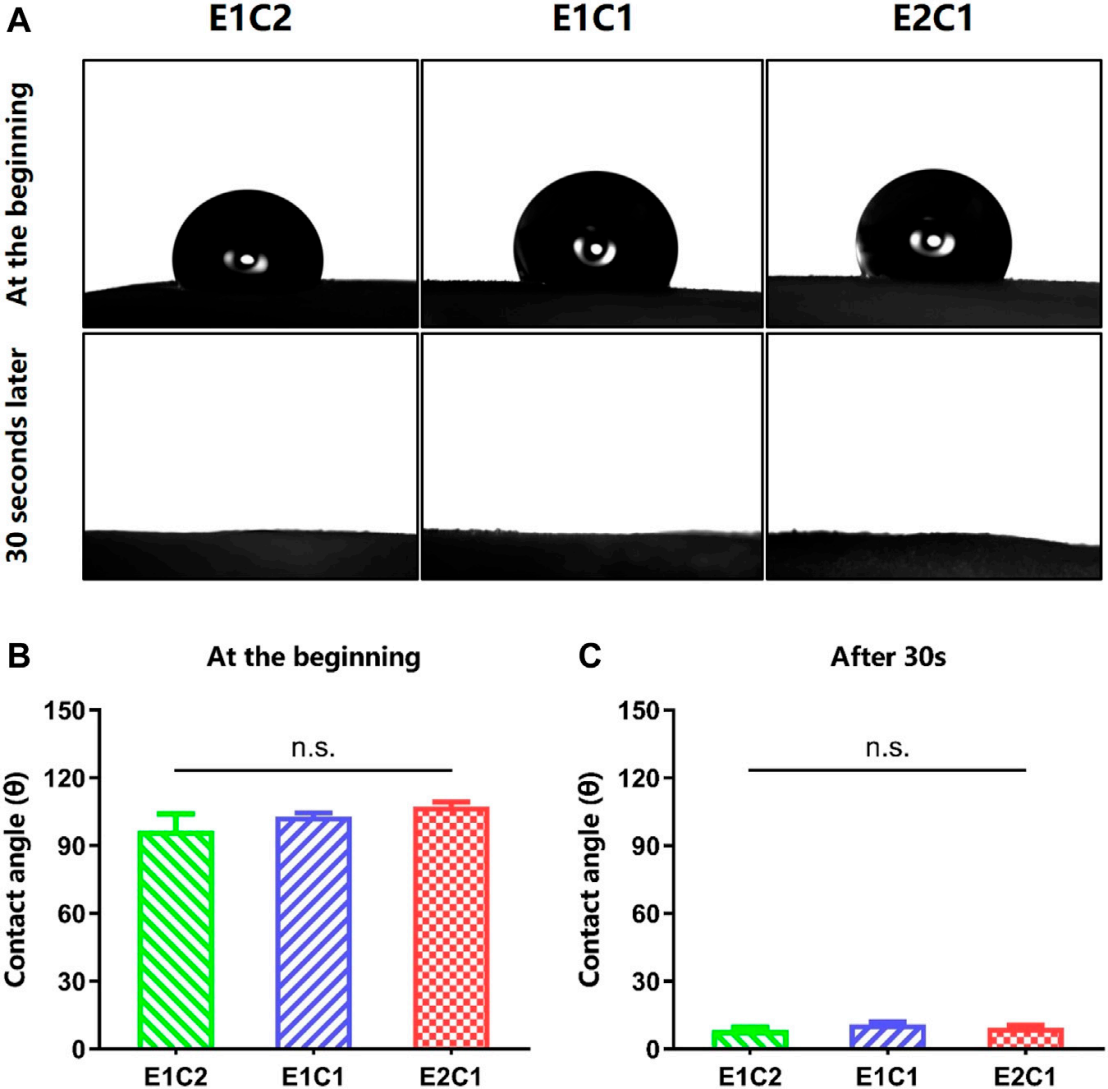
Contact angle of the scaffolds. **(A)** The pictures of the contact angle at the beginning and 30 s later. **(B)** The statistical result of the contact angle at the beginning. **(C)** The statistical result of the contact angle 30 s later. n.s.: *p* > 0.05.

The surface hydrophilicity results of the three groups of NDM-CS scaffolds are shown in Figure 7. The contact angles of droplets were measured when they touched the surface of the scaffolds at the beginning and 30 s later (Figure 7A). When the droplets contacted the surface of the NDM-CS scaffolds, they showed a round spherical shape. However, they were not stable after contact with NDM-CS interestingly. The shape of the droplets changed from round spheres to hemispheres and eventually spreads flat on the scaffold surface. The change in droplet shape indicated that the liquid gradually infiltrated the surface of the material. The contact angle also became significantly smaller during this gradual change. In about 30 s, all droplets on the scaffolds were almost infiltrated into the material, and the contact angle decreased significantly. The contact angle was counted for each of the two states of the droplets. It could be found that the contact angles of all three groups were greatly larger than 90 degrees at the beginning (Figure 7B). The contact angles of E1C2, E1C1 and E2C1 were 96.57° ± 7.457°, 103° ± 1.537° and 107.3° ± 2.051° respectively, and there was no difference between the three groups. This indicated that the surfaces of the three NDM-CS scaffolds were hydrophobic at first. However, the contact angle decreased significantly in 30 s which was about 10 degrees in all three groups (Figure 7C). At this time, the contact angles of E1C2, E1C1 and E2C1 became 8.3° ± 1.435°, 10.97° ± 1.139° and 9.467° ± 1.048° respectively. This demonstrated that the surfaces of the three scaffolds were hydrophilic at this time. This phenomenon may be caused by the surface microstructure and roughness of the freeze-dried NDM-CS scaffolds. The experimental results showed that the NDM-CS composite material was hydrophilic inherently. However, NDM-CS scaffold became rough and formed a microstructure with multiple voids after freeze-drying, which affected the spread of droplets. As time went by, the droplets gradually infiltrate the scaffolds and the contact angle became smaller. The suitable hydrophilicity of the scaffolds could facilitate cell adhesion, extension, nutrient penetration and factor diffusion, so as to better support the growth of cells (Kang et al., 2021).

### Antibacterial Ability of Scaffolds

**FIGURE 8:**
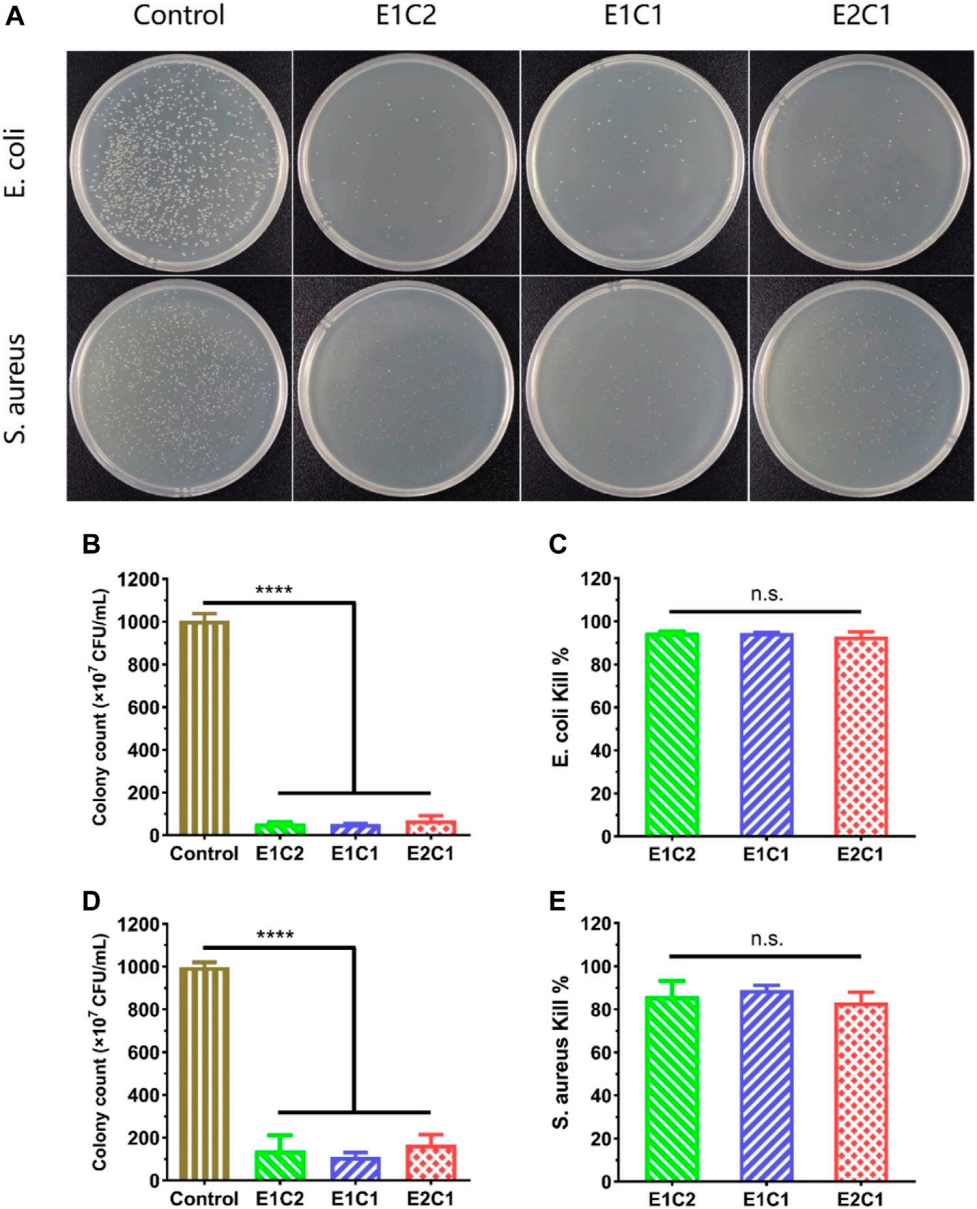
The antibacterial activity of the NDM-CS scaffolds. **(A)** Images of colonies of *E. coli* and *S. aureus* derived from the scaffolds. **(B)** The colonies of *E. coli* in different groups. **(C)** *E. coli* inhibition rate of NDM-CS scaffolds. **(D**) The colonies of *S. aureus* in different groups. **(E)** *S. aureus* inhibition rate of NDM-CS scaffolds.****p < 0.0001. n.s.: *p* > 0.05.

Antimicrobial activity is necessary for nerve scaffolds because bacterial infection interferes nerve repair after nerve injury. The antibacterial neural scaffold could promote recovery by avoiding the inflammatory response after infection. The antibacterial activity of NDM-CS hybrid scaffold was tested by Gram-negative *E. coli* and Gram-positive *S. aureus* (Figure 8). Figure 8A shows the results of colony formation (CFU) on the medium after incubation of *E. coli* and *S. aureus* with the NDM-CS scaffold for 4 h respectively. It was shown that dense and obvious colonies of *E. coli* and *S. aureus* were formed on the plate compared with the blank control group. The number of colonies in the three groups of NDM-CS scaffolds was greatly reduced. Figures 8B,D respectively display the number of colonies of *E. coli* and *S. aureus* in different groups. There was an obvious difference between the number of colonies in the NDM-CS scaffold groups and the blank control group. Figures 8C,E show statistics of the antibacterial rates of NDM-CS scaffold on *E. coli* and *S. aureus* respectively. Among them, the antibacterial rate of the three NDM-CS scaffolds to *E. coli* could reach more than 90%. In addition, the antibacterial rate of NDM-CS stent to *S. aureus* could reach more than 80%. The above results showed that the NDM scaffolds containing CS had a significant antibacterial activity for both *E. coli* and *S. aureus*. This confirmed that CS has a strong antibacterial effect in the scaffolds. The results were consistent with other related studies (Kong et al., 2020). And the inhibition effect of NDM-CS scaffold on *E. coli* and *S. aureus* did not decrease with the decrease of CS’s concentration.

### Cytotoxicity of Scaffolds

**FIGURE 9:**
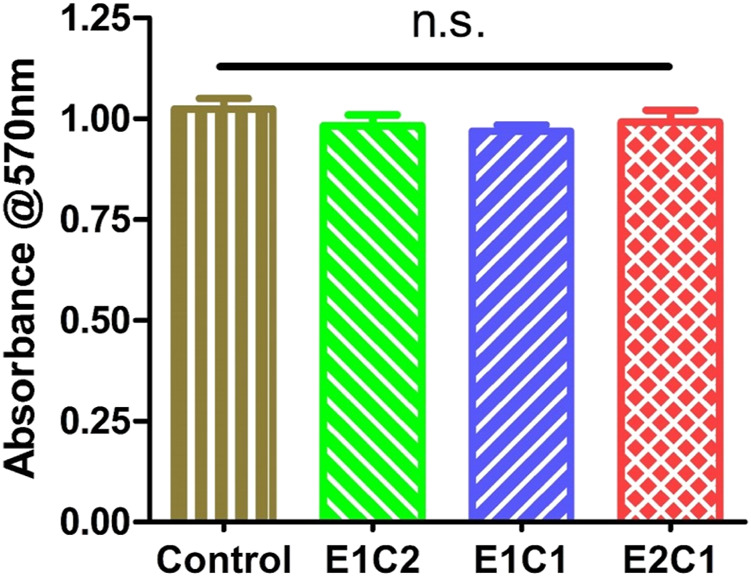
The absorbance of the supernatant at 570 nm of the scaffolds. n.s.: *p* > 0.05.

Figure 9 showed the MTT results for the three NDM-CS scaffold groups 1 day after. The Control group was the normal cell group without material. The absorbance of all three groups of hybrid scaffolds was close to the control group and showed no difference. It also indicated that all the hybrid scaffolds had no toxicity to the cells and could provide a safe growth environment to the cells. The results of the MTT test proved that the NDM-CS scaffolds had excellent biocompatibility.

### Effect of NDM-CS Scaffold on the Growth of Schwann Cells

**FIGURE 10:**
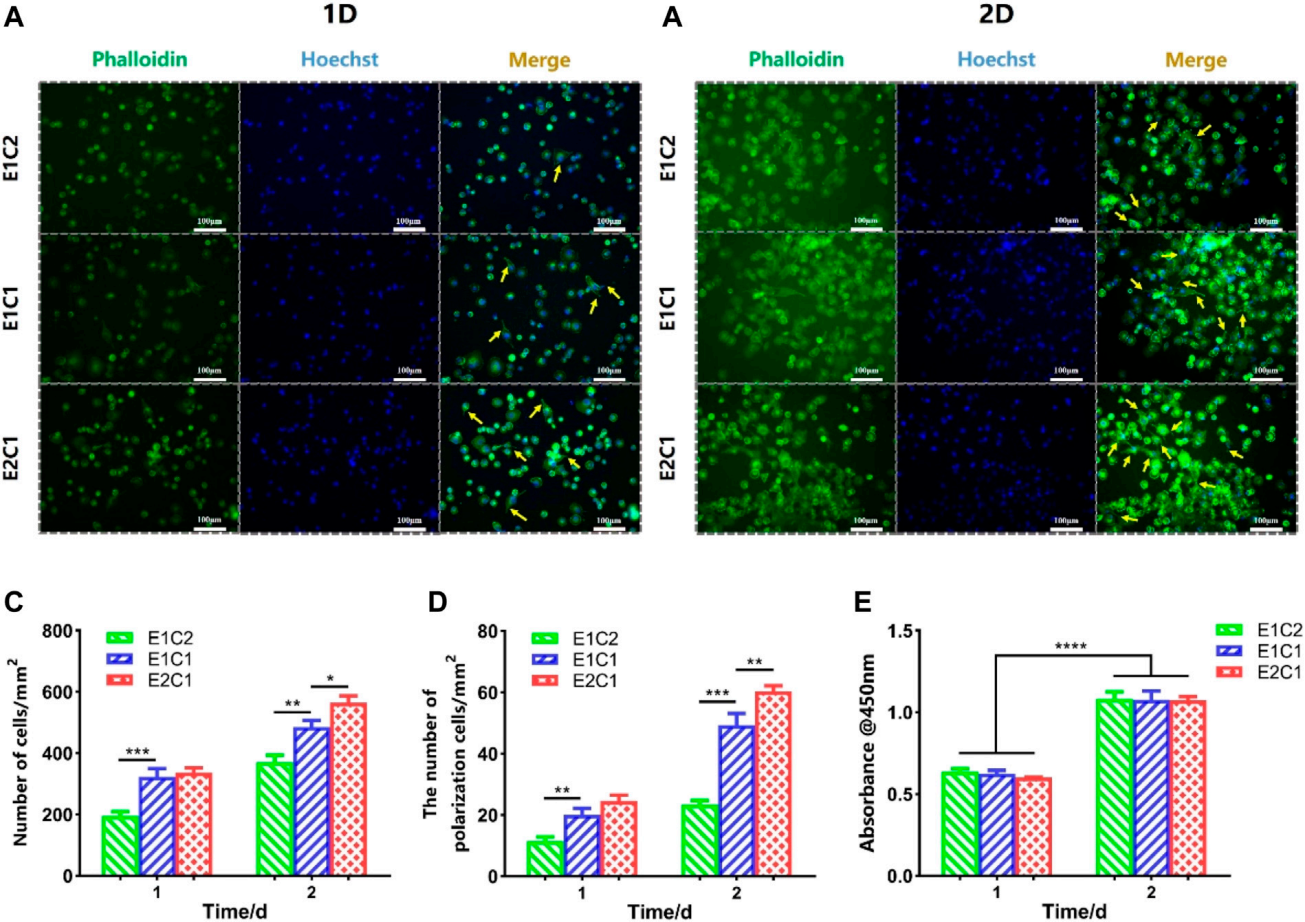
Growth of RSC96 cells under the influence of NDM-CS scaffolds. Immunofluorescence staining of RSC96 cells cultured for **(A)** 1 day and **(B)** 2 days. **(C)** The number of Schwann cells in different groups. **(D)** The number of polarized cells in different groups. **(E)** Cell viability of Schwann cells on different scaffolds of 1 and 2 days. Scale bars represent 100 μm*p < 0.05, **p < 0.01, ***p < 0.001, ****p < 0.0001.

The effect of NDM-CS scaffolds on nerve repair was evaluated by the morphology of Schwann cells. The Schwann cells were cultured with the extract medium of the NDM-CS scaffolds, and the growth of Schwann cells was observed by immunofluorescence staining (Figure 10). Figures 10A,B showed the growth of Schwann cells under the influence of different NDM-CS scaffolds for 1 and 2 days respectively. It was shown that Schwann cells grew vigorously in all groups. And the number of Schwann cells labeled by green fluorescently increased after 2 days of cultured compared to that at 1 day. Figure 10C presents the number of Schwann cells in all groups. Schwann cells were much more in the E1C1 and E2C1 groups than in the E1C2 group. And the number of Schwann cells in the E2C1 group was significantly higher than in both other groups after 2 days. NDM could promote cell proliferation significantly (Chen et al., 2020b) so that the number of Schwann cells in the experimental group increased. Some of the Schwann cells in the experimental groups also showed polarization during the culture process which was also caused by NDM (Agmon and Christman, 2016). These polarized Schwann cells grew pseudopods to varying degrees (indicated by yellow arrows). The number of polarized Schwann cells in all NDM-CS scaffolds was counted in Figure 10D. The trend of Schwann cell polarization was consistent with the change in quantity. The polarized number of Schwann cells was highest in the E2C1 group and was lowest in E1C2. In particular, polarized Schwann cells were much more in the E2C1 group than in the other groups after 2 days. Besides, the viability of Schwann cells was measured under the influence of NDM-CS scaffolds by CCK8 assay. Figure 10E showed the activity of Schwann cells on different NDM-CS scaffolds after culture for 1 and 2 days. There was essentially no difference between the cell densities in each group of scaffolds either 1 or 2 days. However, the OD values in all scaffolds increased significantly after 2 days of incubation which indicated that all NDM-CS scaffolds promoted the growth and proliferation of Schwann cells. However, there was no difference in cell activity between the three groups of scaffolds as the NDM content increased. Therefore, the NDM-CS scaffold could provide a good growth environment for Schwann cells and is suitable for the growth of Schwann cells. NDM-CS scaffold also has a significant promotion effect on the proliferation and polarization of Schwann cells which enhanced with the increase of NDM content.

### The Effect of NDM-CS Scaffolds on Schwann Cell Migration

**FIGURE 11:**
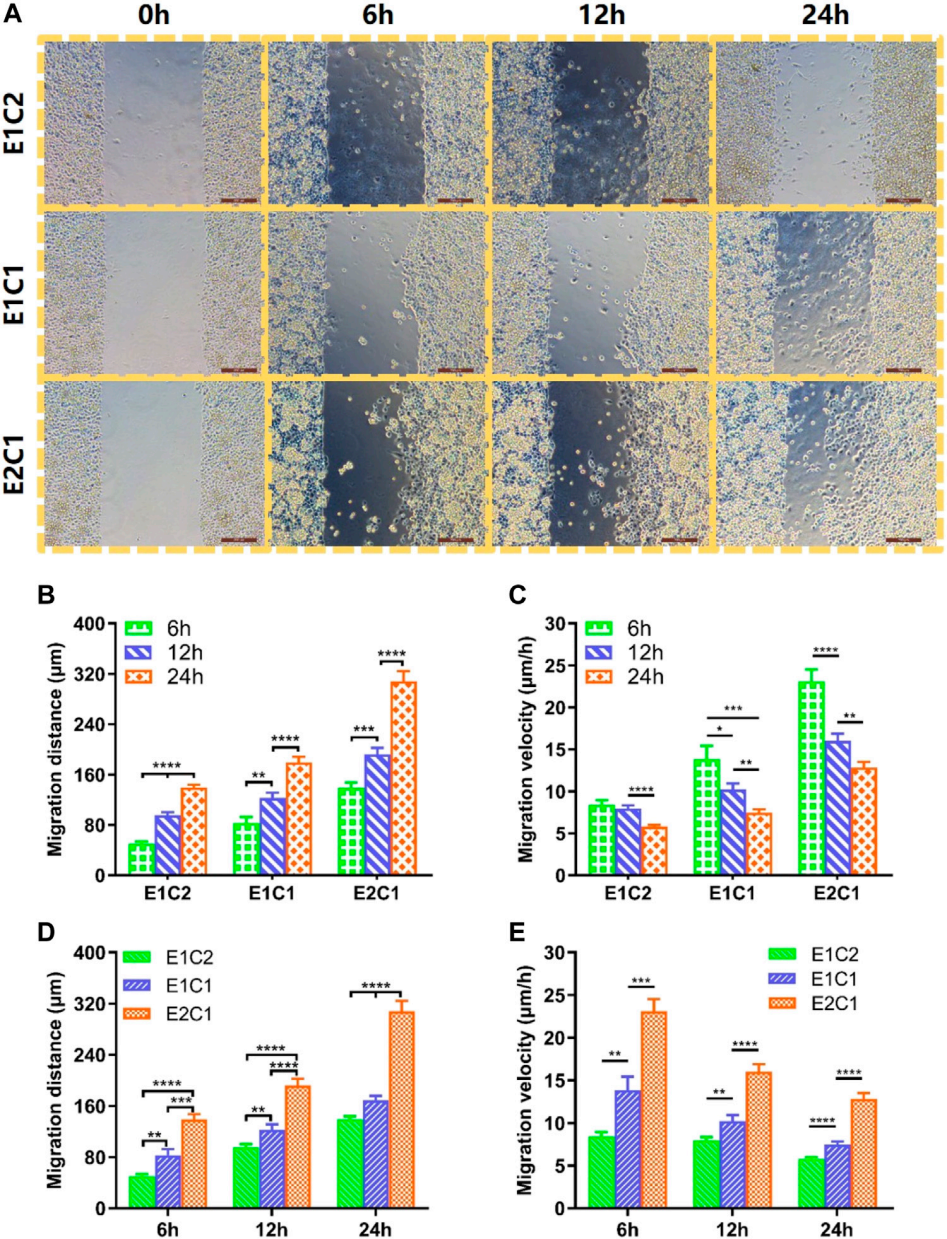
Cell migration of RSC96 cells on cell culture plate surface with different scaffolds extract cultures. **(A)** The migration state of Schwann cells of all groups at different time points (scale bar: 200 μm). **(B)** Changes in migration distance of Schwann cells in different groups. **(C)** Changes in migration velocity of Schwann cells in different groups. **(D)** Migration distance statistics of Schwann cells in the three groups at 6, 12 and 24 h. **(E)** Migration velocity statistics of Schwann cells in the three groups at 6, 12 and 24 h *p < 0.05, **p < 0.01, ***p < 0.001, ****p < 0.0001.

It was proved that the NDM-CS hybrid scaffold could promote the migration of Schwann cells in this study. Figure 11 shows the migration of three groups of Schwann cells at different time. At 0 h, the cell gaps of equal distance were shown in all groups. As time progressed, Schwann cells in all groups migrated toward the center of the gap. The longer the time, the longer distance of Schwann cell migration. The most pronounced migration of Schwann cells was observed in the E2C1 group. The migration distance and migration speed of Schwann cells at different time points were counted in Figures 11A,B respectively. The results showed that the migration distance of Schwann cells increased obviously in all groups with time elapsing. This indicated that the NDM-CS scaffolds have the ability to promote the migration of Schwann cells regardless of the ratio of NDM and CS. However, the migration rate of Schwann cells in each group at different time points showed an opposite trend to the migration distance. The migration velocity of Schwann cells was decreasing in all groups which indicated that the NDM-CS scaffold had the strongest ability to promote Schwann cell migration in the early stage. Although the migration velocity decreased, the Schwann cells were still able to reach a velocity greater than 5 μm/h after 24 h.

We further compared the differences between the different groups (Figures 11C,D). It was found that E1C2 showed the smallest migration distance at all points among the three groups. Meanwhile, the E2C1 group showed the largest cell migration distance at all points. And the migration distance of E2C1 could reach 308.3 μm, which was as twice times as other two groups. In addition, the migration speed of cells in three groups showed a consistent trend. The migration velocity of cells in the E2C1 group was faster than that of E1C1 and E1C2 all the time. The migration velocity of the E2C1 group could reach a maximum value of 23.1 μm/h at 6 h throughout the experiment. Even though the migration velocity was decreasing gradually, the migration velocity of E2C1 could still reach 12.8 μm/h at 24 h. Based on the above results, it revealed that the E2C1 scaffold has the strongest ability to promote the migration of Schwann cells in this study which was attributed to the effect of NDM (Mao et al., 2020). Therefore, NDM-CS as a neural scaffold could provide a suitable growth microenvironment for Schwann cells to migrate. It could be predicted that NDM-CS scaffold would promote the migration of Schwann cells after neurological defects, thus establishing connections between the damaged ends more quickly and building a bridge for neuronal cell regeneration.

## Conclusion

In this study, a composite scaffold with high antimicrobial properties and high biocompatibility was successfully prepared by combining the advantages of nerve decellularized matrix and chitosan. The NDM-CS scaffold exhibited excellent three-dimensional pore structure and hydrophilicity. The CS component in the composite scaffold conferred excellent antibacterial properties to the composite scaffold, avoiding cell invasion and infection and providing a clean microenvironment for nerve repair. In addition, the addition of NDM could significantly promoted the proliferation, polarization and migration of Schwann cells. Moreover, this ability of NDM-CS scaffold was enhanced with the increase of NDM content. Among them, the E2C1 group was considered to be the most excellent performer. It has superior antibacterial property against both *E. coli* and *S. aureus* comparable to E1C2 and E1C1. Meanwhile, E2C1 has the best ability to promote Schwann cell proliferation and migration. All these properties of NDM-CS scaffolds provided a more reliable microenvironment for the neural regeneration process and may have potential application prospects for future clinic application.

## Data Availability

The raw data supporting the conclusion of this article will be made available by the authors, without undue reservation.
